# Effect of Early and Delayed Commencement of Paricalcitol in Combination with Enalapril on the Progression of Experimental Polycystic Kidney Disease

**DOI:** 10.3390/jcdd8110144

**Published:** 2021-10-29

**Authors:** Priyanka S. Sagar, Sayanthooran Saravanabavan, Alexandra Munt, Annette T. Y. Wong, Gopala K. Rangan

**Affiliations:** 1 Michael Stern Laboratory for Polycystic Kidney Disease, Westmead Institute for Medical Research, The University of Sydney, Sydney, NSW 2145, Australia; priyanka.sagar@sydney.edu.au (P.S.S.); sayan.saravanabavan@sydney.edu.au (S.S.); alexandra.munt@sydney.edu.au (A.M.); annette.wong@sydney.edu.au (A.T.Y.W.); 2 Department of Renal Medicine, Westmead Hospital, Western Sydney Local Health District, Sydney, NSW 2145, Australia

**Keywords:** polycystic kidney disease, paricalcitol, vitamin D receptor agonists

## Abstract

Vitamin D secosteroids are intranuclear regulators of cellular growth and suppress the renin-angiotensin system. The aim of this study was to test the hypothesis that the vitamin D receptor agonist, paricalcitol (PC), either alone or with enalapril (E) (an angiotensin-converting enzyme inhibitor), reduces the progression of polycystic kidney disease. Preventative treatment of Lewis polycystic kidney (LPK) and Lewis control rats with PC (0.2 μg/kg i.p. 5 days/week) or vehicle from postnatal weeks 3 to 10 did not alter kidney enlargement. To evaluate the efficacy in established disease, LPK rats received either PC (0.8 μg/kg i.p; 3 days/week), vehicle, E (50 mg/L in water) or the combination of PC + E from weeks 10 to 20. In established disease, PC also did not alter the progression of kidney enlargement, kidney cyst growth or decline in renal function in LPK rats. Moreover, the higher dose of PC was associated with increased serum calcium and weight loss. However, in established disease, the combination of PC + E reduced systolic blood pressure and heart-body weight ratio compared to vehicle and E alone (*p* < 0.05). In conclusion, the combination of PC + E attenuated cardiovascular disease but caused hypercalcaemia and did not alter kidney cyst growth in LPK rats.

## 1. Introduction

Polycystic kidney diseases (PKD) are a group of inherited conditions associated with the development of numerous enlarging renal cysts that lead to life-threatening end-stage kidney disease (ESKD) [1]. Autosomal dominant polycystic kidney disease (ADPKD) is the most prevalent form of PKD in adults and affects 6.5 million people worldwide [1]. Early-onset hypertension occurs prior to the onset of renal impairment and, in addition to left ventricular hypertrophy, contributes to increased mortality, with cardiovascular disease as the most common cause of death for ADPKD patients [2,3,4,5]. In ADPKD patients, hypertension presents on average in their mid-thirties, and they have a greater prevalence of cardiovascular risk factors compared to the general CKD population [5]. Current medical management of ADPKD is focused on the treatment of hypertension with renin-angiotensin inhibitors, such as angiotensin-converting enzyme inhibitors or angiotensin receptor blockers, along with dietary and lifestyle modification to reduce sodium intake and maintain a healthy body mass index, however these interventions only partially slow disease progression [4,6]. Recently, the vasopressin receptor-2 antagonist, tolvaptan, was introduced as the only disease modifying drug for ADPKD, but its universal implementation is limited by off-target adverse effects that include polyuria and idiosyncratic hepatotoxicity. Hence additional pharmacological approaches for PKD that have minimal adverse effects and utilise re-purposed medicines are needed [1,6,7].

The vitamin D group of fat-soluble secosteroids (D_1_–D_5_), are widely utilised in chronic kidney disease (CKD) for the management of secondary hyperparathyroidism. Aside from their classical function in maintaining calcium homeostasis, the vitamin D secosteroids are also intranuclear regulators of cellular growth and differentiation [1,8,9,10] and in vivo have been found to have additional renal- and vascular-protective properties, including the suppression of inflammation, fibrosis [11,12,13,14,15,16], and blood pressure [1,8,13]. In this regard, vitamin D receptor knockout mice develop severe hypertension [17] and, conversely, vitamin D receptor agonists (VDRAs) such as paricalcitol lower blood pressure in conjunction with inhibitors of the renin-angiotensin-aldosterone system (RAAS) in experimental CKD [18]. Data on the role of the vitamin D system on the progression of PKD are limited. In a hypertensive chronic model of PKD, the Lewis Polycystic Kidney Disease (LPK) rat model, chronic vitamin D deficiency exacerbated hypertension (as expected) but mildly reduced kidney cyst growth, suggesting that the vitamin D system may have divergent effects in PKD [19].

Paricalcitol (19-nor-1,25-dihydoxy vitamin-D_2_) is 1000-fold more potent than calcitriol but carries a similar risk of adverse effects, such as hypercalcemia [20]. Based on previous studies in the LPK rats, we hypothesised that paricalcitol may reduce blood pressure and proteinuria. However, as chronic vitamin D deficiency caused a mild reduction in kidney cyst growth in LPK rats, it was important to evaluate whether paricalcitol exacerbated kidney cyst growth. To our knowledge the role of VDRAs in PKD has not been previously investigated [21]. Therefore, the aim of this study was to investigate whether VDRAs have therapeutic effects in PKD and to test the hypothesis that VDRAs reduce the progression of proteinuria and hypertension in the Lewis Polycystic Kidney Disease rat model of PKD (LPK rat) [19]. To test this hypothesis, two sequential chronic experiments were performed to evaluate the preventative (early treatment) and therapeutic (delayed treatment) effects of paricalcitol on the progression of PKD.

## 2. Materials and Methods

### 2.1. Animals

Animals were housed at the animal research facility in the Institute for Clinical Pathology at Westmead Hospital under standard conditions (artificial light, light-dark cycle 1800-0600) with access to water and standard rat chow ad libitum [19]. LPK and Lewis/SSN rats were obtained from the breeding colony at Westmead Hospital [19]. The LPK rat model is a genetic ortholog of human nephronophthisis (NPHP)-9 but phenotypically resembles human autosomal recessive PKD with rapid early and marked cystogenesis between postnatal weeks 3 to 10, the onset of hypertension at week 6, followed by progressive cystic related tubulointerstitial disease leading to renal failure and death soon after week 20 [22,23,24]. All experiments were conducted according to the Australian Code for the Care and Use of Animals for Scientific Purposes [25], and the study ethics protocol was approved by the Western Sydney Local Health District Animal Ethics Committee (Protocol number 4100).

### 2.2. Experimental Design

To determine the effects of early and delayed treatment paricalcitol on the progression of early and established PKD in the LPK rat, two experiments were performed. In Experiment 1, LPK (*n* = 16) and Lewis (*n* = 6) rats were treated from postnatal week 3 until week 10 with either vehicle (V) or paricalcitol (PC) at 0.2 μg/kg administered intraperitoneally (i.p.) 5 days/week with the dose determined by previous studies in rat models of CKD, to minimise the risk of any adverse effects on serum calcium elevation [26]. Rats were weighed daily and the average weight per group was used to determine the dose of paricalcitol. At week 10, rats were placed in metabolic cages for 16 hours for urine collection and sacrificed the following day.

The design of Experiment 2 was based on the results of Experiment 1 and aimed to determine whether a higher dose of PC together with a combination of angiotensin blockade had efficacy in established disease, based on previous studies in models of hypertension and CKD [11,27]. Enalapril, an angiotensin-converting enzyme inhibitor, was used for renin-angiotensin blockade. In Experiment 2, LPK and Lewis rats were treated with either vehicle (V), paricalcitol (PC; 0.8 μg/kg i.p.; 3 days/week), enalapril (E; 50 mg/L in tap water), or a combination of both paricalcitol and enalapril (PC + E) from postnatal week 10 until week 20, with dosing based on previous studies [11,15,28,29]. As mentioned, the dose and frequency of PC was modified in Experiment 2 (compared to Experiment 1) based on previous studies to determine if a higher weekly dose had an effect on kidney enlargement and also to evaluate the effects on blood pressure and proteinuria [11,15]. Rats were weighed weekly and water intake was measured twice a week (as previously described [24]) to ensure that the amount of E consumed was similar between combined and single treatment groups. Rats were placed in metabolic cages for 16 hours at weeks 13, 16 and 19 to collect urine for analysis of renal function. At sacrifice, rats were anaesthetized by an intraperitoneal injection of ketamine/xylazine, blood was collected by cardiac puncture, and both kidneys and heart were removed by surgical dissection as described in previous studies [23].

### 2.3. Assessment of Serum Calcium, Phosphate, Albumin and Renal Function

Serum 25-hydroxy (OH) vitamin (Experiment 1 only), corrected calcium, phosphate, creatinine and albumin were analyzed at the Institute for Clinical Pathology and Medical research (ICPMR) at Westmead Hospital, as previously described [19]. In Experiment 2, urine volume, proteinuria, urine calcium and creatinine were additionally analyzed and creatinine clearance was calculated and corrected for body surface area [19].

### 2.4. Tail-Cuff Systolic Blood Pressure

Serial measurements of tail-cuff systolic blood pressure were undertaken non-invasively in conscious rats using a tail-sensor (MacLab, AD Instruments) and tail-cuff inflation at week 13, 16 and 19 as previously described [23]. The systolic blood pressure was defined as the appearance of the tail arterial pulse wave with cuff deflation. Five measurements were undertaken at each session for each rat and the mean calculated.

### 2.5. Histology

For experiment 2, coronal slices of kidney and heart were immersion-fixed in methyl carnoy solution and embedded in paraffin. Tissue sections, cut at 4 microns in thickness, were stained with Periodic-acid Schiff. Because cystic kidney disease in the LPK rat at week 20 is diffuse and very severe, only qualitative analyses of the sections were performed.

### 2.6. Statistical Analysis

The data was entered into JMP Pro statistical software (Version 15.2, SAS institute) and Prism (version 9.1.0, GraphPad) and presented as mean and standard deviation. Comparison between groups was performed by ANOVA followed by post-hoc analysis with Tukey–Kramer honestly significant difference test. A *p* value less than 0.05 was defined as statistically significant.

## 3. Results

### 3.1. Effect of Early Treatment with Paricalcitol on Disease Progression in LPK Rats

#### 3.1.1. Renal function, Proteinuria and Serum Calcium

There were no deaths or adverse events. At Week 10, LPK + V rats developed increased proteinuria and urine volume, and impairment of endogenous creatinine clearance without significantly increased serum creatinine compared to the Lewis + V rats, and this was not affected by treatment with PC (Table 1). There was also no change in serum calcium in PC treated rats at week 10 compared with V (Table 1). There was a significant reduction in 25, hydroxy-vitamin D in both LPK rat groups compared with Lewis rats, as expected with early kidney disease (*p* < 0.01, Table 1).

#### 3.1.2. Renal and Cardiac Enlargement

The two-kidney weight to body weight ratio is a surrogate marker of cyst growth and increased 6-fold in LPK + V rats compared to the Lewis + V rats at week 10. Treatment with PC did not alter the progression of kidney enlargement (Table 2). Cardiac enlargement, as determined by the heart-to-body weight ratio which is a surrogate marker for left ventricular hypertrophy in LPK rats, was 1.3-fold higher in LPK + V rats compared to the Lewis + V rats and was not affected by PC treatment (Table 2).

### 3.2. Effect of Delayed Treatment with Paricalcitol on Disease Progression in LPK Rats

#### 3.2.1. General Health of LPK and Lewis Rats

**Figure 1:**
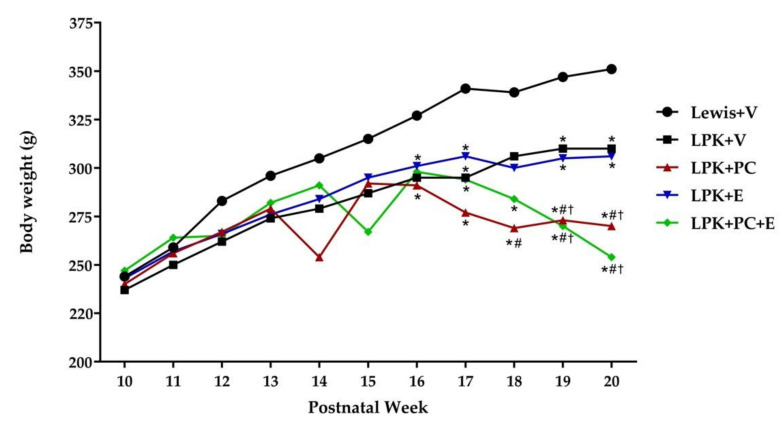
Body weight over time in late PKD. Abbreviations: LPK, Lewis polycystic kidney rat; V, vehicle; PC, paricalcitol; E, enalapril; g, grams. Data represented as mean. * *p* = <0.05 compared to Lewis + V group, # *p* < 0.05 compared to the LPK + V group. † *p* < 0.05 compared to the LPK + E group.

Two LPK rats died in the study. At week 18, one rat in the LPK + V group developed hemiplegia and was suspected to have suffered a cerebrovascular event and was euthanized early. At week 19, another rat in the LPK + PC was found dead, with autopsy showing the likely cause of death was ESKD. Both rats were excluded in the final timepoint analyses. There was no difference in body weight between Lewis and LPK rats treated with either V, PC, E or PC + E up to week 16. From week 16 onwards, LPK rats from all groups were smaller than the PKD-unaffected Lewis rats. LPK rats treated with PC alone or in combination with E consistently had lower body weights from week 18 onwards (Figure 1, Table S1).

#### 3.2.2. Water Intake and Urine Output in Lewis and LPK Rats

Water intake was increased in all LPK rat groups compared to Lewis + V (Table S2). At week 13, 16 and 19, LPK rats urine output was significantly increased compared to Lewis rats, as expected, probably due to loss of renal concentrating ability (Table S3). There was no change in urine output between LPK groups (Table S3).

#### 3.2.3. Renal Function, Serum Calcium, Serum Phosphate and Urinary Calcium Excretion

At week 20 renal function, as determined by both serum creatinine and endogenous creatinine clearance, was significantly impaired in LPK rats compared to the Lewis + V and this was not affected by treatment with PC, E or a combination of PC + E (Table 3). At Week 20, LPK rats receiving PC developed a 1.3-fold increase in serum calcium compared to the LPK + V group (*p* < 0.01, Table 3). Serum phosphate was elevated in LPK + PC + E rats compared to LPK + V and LPK + E groups, but there was no significant difference between any other group (Table 3). The urinary calcium to creatinine ratio was increased in all LPK groups compared to Lewis controls at week 13 and 19 (Table S4). At week 19, the urinary calcium to creatinine ratio was increased in LPK + PC compared with LPK + V and LPK + E (Table S4).

#### 3.2.4. Progression of Proteinuria

**Figure 2:**
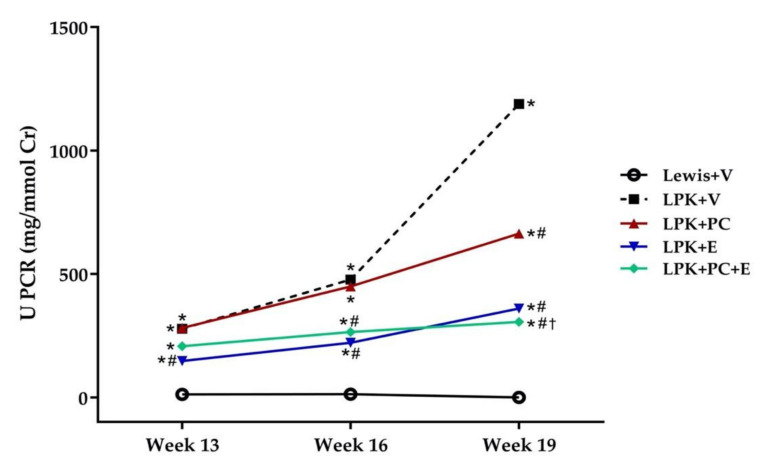
Effect of delayed paricalcitol treatment on the progression of proteinuria in LPK rats. Abbreviations: U PCR, Urine protein creatinine ratio measured in mg/mmol creatinine; LPK, Lewis polycystic kidney rat; V, vehicle; PC, paricalcitol; E, enalapril. Data represented as mean. * *p* < 0.05 compared to Lewis rat, # *p* < 0.05 vs. LPK + V group, † *p* < 0.05 compared to LPK + PC group.

The LPK + V group developed significant proteinuria compared to the Lewis + V group and this increased between weeks 13 and 19 (Figure 2, Table S5). Treatment with PC alone caused a delayed reduction in proteinuria by 41% which was detected only at week 19 compared to LPK + V. In contrast, continuous treatment of LPK rats with E consistently reduced the progression of proteinuria by 46.9%, 53.7% and 69.0% on weeks 13, 16, and 19, respectively. This reduction was also detectable with the combination of PC + E at weeks 16 and 19, however, the combination of PC + E had no additional anti-proteinuric effect when compared to E alone (*p* = 0.09, Figure 2, Table S5).

#### 3.2.5. Kidney Enlargement and Cyst Growth

The 2KW:BW ratio, which is a surrogate measure of kidney cyst growth in LPK rats, was increased 11.8-fold in the LPK + V group compared to the Lewis + V rats, and delayed treatment with PC did not affect this increase (Table 4). By light microscopy, LPK rats developed diffuse and advanced cystic kidney disease with little normal renal parenchyma, and the progression of these changes was not affected by treatment with either PC, E or PC + E (Figure S1). In addition, there were focal areas of intra-tubular crystals present in all LPK groups (Figure S2).

#### 3.2.6. Progression of Hypertension and Cardiac Enlargement

**Figure 3:**
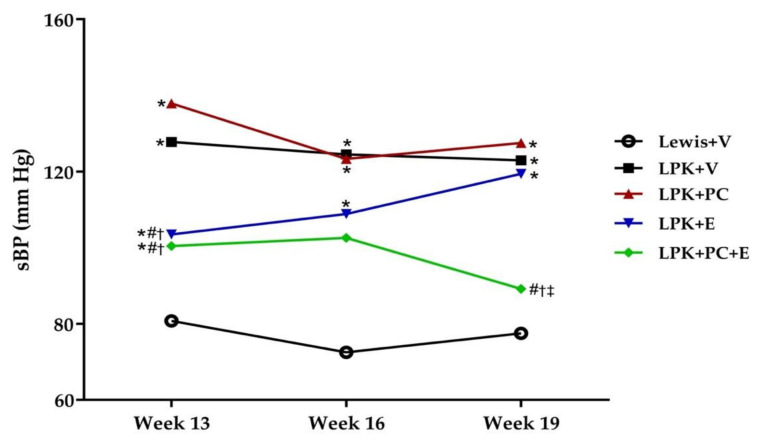
Effect of delayed paricalcitol treatment on the progression of hypertension in LPK rats. sBP, systolic blood pressure; LPK, Lewis polycystic kidney rat; V, vehicle; PC, paricalcitol; E, enalapril. Data represented as mean. * *p* < 0.05 compared to Lewis + V; # *p* < 0.05 compared to LPK + V. † *p* < 0.01 compared to LPK + PC. ‡ *p* < 0.01 compared to LPK + E.

Tail-cuff systolic blood pressure increased in LPK + V rats between weeks 13 and 19 compared to the Lewis + V groups (Figure 3, Table S6). Treatment with PC alone did not alter the progression of hypertension in LPK rats (Figure 3, Table S6). In contrast, treatment with enalapril reduced systolic blood pressure at week 13 (*p* = 0.0073 compared with LPK + V) but this was not sustained for the remainder of the study (*p* = 0.3651 and *p* = 0.9995 at weeks 16 and 19 compared with LPK + V, respectively, Figure 3, Table S6). Moreover, the combination of PC + E led to a sustained attenuation of systolic blood pressure at both week 13 and week 19 compared to LPK + V (*p* = 0.0009 and *p* = 0.0196, respectively) and was not different to Lewis + V group (*p* = 0.1008 and *p* = 0.2987 at week 13 and 19, respectively, Figure 3, Table S6).

The heart-to-body weight (HW:BW) ratio was increased 1.8-fold in the LPK + V group compared to the Lewis + V group. Treatment with either PC or E did not alter the HW:BW ratio compared to LPK + V, whereas the combination of PC + E reduced the HW:BW by 17.8% (*p* = 0.0002, compared to LPK + V rat group; Table 5). By light microscopy, there were no differences between the LPK rat treatment groups in cardiac histology (Figure S3).

## 4. Discussion

In this study we evaluated the effect of early and delayed treatment with paricalcitol on the long-term progression of a hypertensive model of PKD. The results showed that: (i) continuous treatment with paricalcitol either in early or established PKD did not have any disease-modifying effects on renal function or kidney cyst growth; (ii) combination treatment in the late phase of disease led to a sustained reduction in systolic blood pressure and cardiac enlargement to a greater extent than enalapril alone; (iii) paricalcitol reduced proteinuria; however, not to the extent of enalapril therapy; (iv) the above effects of paricalcitol in late disease occurred at the cost of increased serum calcium, weight loss and increase urinary calcium excretion. Taken together, the beneficial effect of PC on blood pressure and proteinuria in LPK rats are similar to that demonstrated in other models of hypertension and CKD but without disease-modifying effects and potential for risk of adverse effects due to elevation in serum calcium [11,30].

Recognition of the non-classical actions of vitamin D metabolites and VDRAs has led to their evaluation as therapeutic agents for a broad range of chronic conditions including cancer [10], cardiovascular disease [31], autoimmune disorders [32] and CKD [33,34]. Several clinical and experimental studies have suggested that VDRAs provide cardiovascular protection and reduce blood pressure and proteinuria [35]. However, knowledge regarding the role of vitamin D signalling in PKD remains incomplete. In LPK rats, chronic dietary deficiency of vitamin D exacerbated proteinuria but unexpectedly caused a small but significant reduction in kidney cyst growth [19]. In contrast, in a small longitudinal cohort study of humans with ADPKD, low levels of 25-OH vitamin D did not predict changes in total kidney volume or blood pressure [36]. An important disease-specific finding of the current study was that neither early nor delayed treatment with paricalcitol altered kidney cyst growth [19].

Hypertension occurs early in PKD due to local hyperactivation of the RAAS with cyst formation [2]. The results of the current study support these findings by showing that treatment with paricalcitol in combination with enalapril reduced blood pressure. The findings demonstrate the known relationship between vitamin D and VDR signalling on reducing the activity of circulating and local tissue activation of RAAS in PKD [37]. The reduction in cardiac enlargement is likely secondary to this sustained blood pressure reduction and its protective effects on left ventricular hypertrophy. Given the significant role of cardiovascular disease in the morbidity and mortality of PKD [5,38], the potential to further enhance the effects of RAAS blockade with VDRAs holds potential, with the caveat of provoking hypercalcemia.

Previous observational data suggested that VDRAs were associated with a benefit on mortality in end-stage kidney failure [39], but caution has been raised in the CKD Stage 3-4 subgroup due to adverse outcomes, particularly hypercalcaemia and augmentation of fibroblast growth factor 23 (FGF23) levels, as reviewed recently [35]. Relevant to the former concern, in the current study, delayed treatment with paricalcitol elevated serum calcium during the period of rapid decline in renal function and markedly reduced body weight gain. The latter was not due to dehydration (as water intake was similar in all LPK groups). Unfortunately, food intake was not measured but we hypothesize that the weight loss was due to anorexia secondary to elevated calcium. Previous studies in non-CKD models have shown that vitamin D analogs cause hypercalcemia and lead to anorexia and weight loss [40,41]. In contrast, the dose regimen has been well tolerated in 5/6 nephrectomised rats [11,12,15,26] suggesting that other factors specific to the LPK disease model may be responsible for the sensitivity to hypercalcemia. In any case, our findings suggest that while paricalcitol may have some beneficial effects in PKD, the use of VDRAs with lower calcaemic-inducing activity may be required in future studies. In addition, given that serum levels of FGF23 are elevated innately in PKD models [42,43] and increased by paricalcitol [30], further understanding of the effects of VDRAs on this endpoint is also needed.

Paricalcitol partially reduced proteinuria compared to control LPK rats. This effect was independent of blood pressure and kidney size as there was no change to either with paricalcitol therapy alone. These findings are consistent with human observational studies and uraemic rat models which show that VDRAs reduce proteinuria, and this may be through inhibition of podocyte injury and preservation of slit diaphragm proteins [44,45,46], in addition to its anti-inflammatory effects [13,14]. Similarly, the combination of paricalcitol and enalapril therapy in 5/6 nephrectomy rats resulted in a marked reduction in inflammation and fibrosis through suppression of RAAS and *TGF-β* gene expression [13]. This anti-inflammatory and anti-fibrotic action is unique to paricalcitol compared with calcitriol, thought to be due to differential gene expression [13,47,48]. In the present study, histological disease in the LPK rats at week 20 showed advanced cystic kidney disease, and there were no differences between the treatment groups. However, these data do not exclude the possibility that glomerular and/or tubulointerstitial disease, either by ultrastructure changes or gene expression, could have been altered at an earlier time point.

The main strength of the current study is that it has examined both early and delayed efficacy of paricalcitol in a robust hypertensive model of PKD. There were some limitations in the current study. First, although the molecular mechanisms of kidney cyst formation share common features, disease-specific phenotypic differences between the various sub-types of PKD may be present. In that regard, in this study, the hypothesis was investigated using a genetic ortholog of NPHP-9 rather than that of ADPKD. It is noteworthy that in a post-hoc analysis of the HALT-PKD consisting of 864 individuals with ADPKD, levels of vitamin D metabolites did not predict either change in Ht-TKV and eGFR decline consistent with the results of this study [42]. In any case, further interventional studies using genetic orthologs of ADPKD are needed to fully evaluate the role of VDRAs. Second, the dose of paricalcitol used in the two experiments was different and may not necessarily be applicable to human disease. In Experiment 2, the dose was increased to 0.8 μg/kg/day three times per week based on previous studies [11,30]. This elevated serum calcium in LPK rats and caused weight loss, and our results cannot completely exclude the possibility that this may have masked a protective effect (if any) on kidney disease. Therefore, further studies investigating the role of other VDRAs with anti-calcaemic activity in in vitro and in vivo genetic ortholog models of ADPKD would be helpful [49]. In addition, it would be interesting to evaluate the effects of combining VDRAs with calcium-sensing receptor agonists, given that they suppress parathyroid hormone-induced augmentation of renal cyclic AMP and have been shown to attenuate kidney enlargement as well as serum calcium [50]. Thirdly, we did not evaluate 1,25-hydroxy vitamin D levels in this study to verify any additional bioactivity of paricalcitol. Finally, it is also possible that higher doses of enalapril may have led to a sustained reduction in blood pressure in the delayed treatment experiment.

## 5. Conclusions

In conclusion, this study shows that paricalcitol in combination with enalapril leads to a sustained reduction in blood pressure and attenuates cardiac enlargement in PKD. Paricalcitol also reduced proteinuria, but this effect was not superior to ACE inhibition. While these results verify that the protective effects of VDRAs observed in generic models of CKD also apply to PKD, neither paricalcitol nor the combination with enalapril altered the progression of kidney cyst growth. In addition, paricalcitol elevated serum calcium and reduced body weight despite being used at doses similar to other CKD models [11,30], adding a significant caveat to its potential clinical application for attenuating cardiovascular disease in PKD.

## Figures and Tables

**Table 1:** Effect of early treatment with paricalcitol on renal function and disease progression at week 10.

	Lewis + V (*n* = 3)	Lewis + PC (*n* = 3)	LPK + V (*n* = 8)	LPK + PC (*n* = 8)
Serum Ca (mmol/L)	2.5 ± 0.1	2.4 ± 0.1	2.3 ± 0.4	2.3 ± 0.2
Serum Albumin (mg/dL)	27 ± 2	27 ± 2	27 ± 4	28 ± 4
Serum Creatinine (μmol/L)	28 ± 2	35 ± 16	38 ± 7	42 ± 5
Serum 25-OH vitamin D	133 ± 9	120 ± 13	82 ± 14 *	95 ± 14 *
Urine volume (mls/16hrs)	5 ± 1	5 ± 1	11 ± 4 *	10 ± 3 *
CrCl (µmol/L/g BW)	7.8 ± 0.9	7.0 ± 2.8	4.5 ± 1.5 *	3.4 ± 1.2 *
Urine PCR (mg/mmol Cr)	5.4 ± 0.7	5.3 ± 0.8	162.7 ± 49.1 *	147.2 ± 62.9 *^1^

^1^ Abbreviations: LPK, Lewis polycystic kidney rat; V, vehicle; PC, paricalcitol; Ca, corrected calcium; 25-OH vitamin D, 25-hydroxy vitamin D; CrCl, endogenous creatinine clearance corrected for body weight (BW); PCR, protein to creatinine ratio. Data expressed as mean ± standard deviation. * *p* < 0.01 compared to Lewis + V.

**Table 2:** Effect of early treatment with paricalcitol on renal and cardiac enlargement at week 10.

	Lewis + V (*n* = 3)	Lewis + PC (*n* = 3)	LPK + V (*n* = 8)	LPK + PC (*n* = 8)
Week 10 BW (g)	282 ± 5	283 ± 11	232 ± 14 *	232 ± 14 *
Right KW (g)	1.1 ± 0.1	1.1 ± 0.1	6.7 ± 0.6 *	6.9 ± 0.9 *
Left KW (g)	1.1 ± 0.1	1.1 ± 0.1	6.9 ± 0.8 *	6.8 ± 0.8 *
2KW:BW ratio (%)	0.8 ± 0.0	0.8 ± 0.0	5.9 ± 0.5 *	5.7 ± 0.5 *
HW (g)	0.83 ± 0.05	0.83 ± 0.04	0.94 ± 0.07 *	1.00 ± 0.05 *
HW:BW ratio (%)	0.29 ± 0.01	0.29 ± 0.03	0.40 ± 0.02 *	0.41 ± 0.03 *^1^

^1^ Abbreviations: LPK, Lewis polycystic kidney rat; V, vehicle; PC, paricalcitol; BW, body weight; KW, kidney weight; HW, height weight. Data expressed as mean ± standard deviation. * *p* < 0.01 compared to Lewis + V.

**Table 3:** Effect of delayed treatment of paricalcitol, enalapril and combination therapy on renal function and serum calcium in LPK rats at week 20.

	Lewis + V (*n* = 6)	LPK + V (*n* = 7)	LPK + PC (*n* = 6)	LPK + E (*n* = 7)	LPK + PC + E (*n* = 7)
Serum Ca (mmol/L)	2.7 ± 0.0	2.7 ± 0.1	3.6 ± 0.1 *	2.8 ± 0.0	3.6 ± 0.1 *
Serum PO_4_ (mmol/L)	2.2 + 0.4	2.0 ± 0.2 ^#^	2.1 ± 0.2	2.1 ± 0.2 #	2.6 ± 0.5
Serum Cr (μmol/L)	32 ± 2	144 ± 33 *	136 ± 15 *	124 ± 16 *	164 ± 41 *
CrCl (µmol/L/g BW)	8.1 ± 1.5	1.4 ± 0.7 *	1.4 ± 0.3 *	1.7 ± 0.2 *	1.4 ± 0.3 *^1^

^1^ Abbreviations: LPK, Lewis polycystic kidney rat; V, vehicle; PC, paricalcitol; E, enalapril; Ca, corrected calcium; PO4, phosphate; CrCl, endogenous creatinine clearance corrected for body weight (BW). Data expressed as mean ± standard deviation. * *p* < 0.01 compared to Lewis + V. ^#^ *p* < 0.05 compared to LPK + PC + E.

**Table 4:** Effect of delayed treatment of paricalcitol, enalapril and combination therapy on renal enlargement LPK rats at week 20.

	Lewis + V (*n* = 6)	LPK + V (*n* = 7)	LPK + PC (*n* = 6)	LPK + E (*n* = 7)	LPK + PC + E (*n* = 7)
Final BW (g)	355 ± 19	302 ± 19 *	266 ± 12 *#	314 ± 19 *	258 ± 238 *#
Right KW (g)	1.13 ± 0.08	10.22 ± 1.79 *	8.57 ± 1.67 *	12.00 ± 1.82 *	9.31 ± 1.60 *
Left KW (g)	1.12 ± 0.05	12.30 ± 1.90 *	10.01 ± 0.46 *	13.0 ± 1.87 *	10.74 ± 2.28 *
2KW:BW (%)	0.63 ± 0.03	7.44 ± 0.70 *	6.99 ± 0.60 *	7.97 ± 0.90 *	7.74 ± 0.86 *

Abbreviations: LPK, Lewis polycystic kidney rat; V, vehicle; PC, paricalcitol; BW, body weight; KW, kidney weight. Data expressed as mean ± standard deviation. * *p* < 0.01 compared to Lewis + V. # *p* < 0.05 compared to LPK + V.

**Table 5:** Effect of delayed treatment of paricalcitol, enalapril and combination therapy on cardiac enlargement LPK rats at week 20.

	Lewis + V (*n* = 6)	LPK + V (*n* = 7)	LPK + PC (*n* = 6)	LPK + E (*n* = 7)	LPK + PC + E (*n* = 7)
HW (g)	0.90 ± 0.05	1.36 ± 0.16 *	1.11 ± 0.12 *#	1.27 ± 0.07 *	0.95 ± 0.12 #
HW:BW (%)	0.25 ± 0.01	0.45 ± 0.03 *	0.42 ± 0.03 *	0.40 ± 0.02 *	0.37 ± 0.03 *#^1^

^1^ Abbreviations: LPK, Lewis polycystic kidney rat; V, vehicle; PC, paricalcitol; BW, body weight; HW, height weight. Data expressed as mean ± standard deviation. * *p* < 0.01 compared to Lewis + V. # *p* < 0.05 compared to LPK + V.

## Data Availability

All data generated and reported in this study have been presented as tables and/or figures within this article.

## Supplementary Materials

The following are available online at https://www.mdpi.com/article/10.3390/jcdd8110144/s1, Table S1: Effect of delayed paricalcitol treatment on body weight (g) over time. Table S2: Mean water intake (mls/day) in LPK and Lewis rats over the treatment period in the delayed paricalcitol treatment. Table S3: Effect of delayed paricalcitol treatment on urine volume (mls) in LPK rats. Table S4: Effect of delayed paricalcitol treatment on urinary calcium excretion (urine calcium:creatinine ratio) in LPK rats. Table S5: Effect of delayed paricalcitol treatment on the progression of proteinuria in LPK rats. Table S6: Effect of delayed paricalcitol treatment on the progression of systolic blood pressure in LPK rats. Figure S1: Effect of delayed treatment (weeks 10 to 20) with paricalcitol, enalapril and combination of paricalcitol with enalapril on progression of cystic kidney disease in LPK rats. Figure S2: Intra-tubular crystal formation in LPK rats at week 20. Figure S3: Effect of delayed treatment (weeks 10 to 20) with paricalcitol, enalapril and combination of paricalcitol with enalapril on cardiac histology in LPK rats.

## References

[1] Rangan G.K., Tchan M.C., Tong A., Wong A.T.Y., Nankivell B.J. (2016). Recent advances in autosomal-dominant polycystic kidney disease. Intern. Med. J..

[2] Ecder T., Schrier R.W. (2009). Cardiovascular abnormalities in autosomal-dominant polycystic kidney disease. Nat. Rev. Nephrol..

[3] Chapman A.B., Torres V.E., Perrone R.D., Steinman T.I., Bae K.T., Miller J.P., Miskulin D.C., Oskoui F.R., Masoumi A., Hogan M.C. (2010). The HALT Polycystic Kidney Disease Trials: Design and Implementation. Clin. J. Am. Soc. Nephrol..

[4] Schrier R.W. (2009). Renal Volume, Renin-Angiotensin-Aldosterone System, Hypertension, and Left Ventricular Hypertrophy in Patients with Autosomal Dominant Polycystic Kidney Disease. J. Am. Soc. Nephrol..

[5] Helal I., Reed B., Mettler P., Mc Fann K., Tkachenko O., Yan X.-D., Schrier R.W. (2012). Prevalence of Cardiovascular Events in Patients with Autosomal Dominant Polycystic Kidney Disease. Am. J. Nephrol..

[6] Chapman A.B., Devuyst O., Eckardt K.-U., Gansevoort R.T., Harris T., Horie S., Kasiske B.L., Odland D., Pei Y., Perrone R.D. (2015). Autosomal-dominant polycystic kidney disease (ADPKD): Executive summary from a Kidney Disease: Improving Global Outcomes (KDIGO) Controversies Conference. Kidney Int..

[7] Torres V.E., Chapman A.B., Devuyst O., Gansevoort R.T., Grantham J.J., Higashihara E., Perrone R.D., Krasa H.B., Ouyang J., Czerwiec F.S. (2012). Tolvaptan in Patients with Autosomal Dominant Polycystic Kidney Disease. N. Engl. J. Med..

[8] Panchapakesan U., Pollock C. (2018). Drug repurposing in kidney disease. Kidney Int..

[9] Reed-Gitomer B. (2013). Vitamin D deficiency: A nontraditional risk factor in polycystic kidney disease?. Am. J. Physiol. Physiol..

[10] Duffy M.J., Murray A., Synnott N., O’Donovan N., Crown J. (2017). Vitamin D analogues: Potential use in cancer treatment. Crit. Rev. Oncol..

[11] Finch J.L., Suarez E.B., Husain K., Ferder L., Cardema M.C., Glenn D.J., Gardner D.G., Liapis H., Slatopolsky E. (2012). Effect of combining an ACE inhibitor and a VDR activator on glomerulosclerosis, proteinuria, and renal oxidative stress in uremic rats. Am. J. Physiol. Physiol..

[12] Husain K., Ferder L., Mizobuchi M., Finch J., Slatopolsky E. (2008). Combination Therapy with Paricalcitol and Enalapril Ameliorates Cardiac Oxidative Injury in Uremic Rats. Am. J. Nephrol..

[13] Martínez-Arias L., Panizo S., Alonso-Montes C., Martín-Vírgala J., Martín-Carro B., Fernández-Villabrille S., Gil-Albert C.G., Palomo-Antequera C., Fernández-Martín J.L., Ruiz-Torres M.P. (2021). Effects of calcitriol and paricalcitol on renal fibrosis in CKD. Nephrol. Dial. Transplant..

[14] Melamed M.L., Thadhani R.I. (2011). Vitamin D Therapy in Chronic Kidney Disease and End Stage Renal Disease. Clin. J. Am. Soc. Nephrol..

[15] Mizobuchi M., Morrissey J., Finch J.L., Martin D.R., Liapis H., Akizawa T., Slatopolsky E. (2007). Combination Therapy with an Angiotensin-Converting Enzyme Inhibitor and a Vitamin D Analog Suppresses the Progression of Renal Insufficiency in Uremic Rats. J. Am. Soc. Nephrol..

[16] Ta M.H., Harris D.C., Rangan G.K. (2013). Role of interstitial inflammation in the pathogenesis of polycystic kidney disease. Nephrology.

[17] Xiang W., Kong J., Chen S., Cao L.-P., Qiao G., Zheng W., Liu W., Li X., Gardner D.G., Li Y.C. (2005). Cardiac hypertrophy in vitamin D receptor knockout mice: Role of the systemic and cardiac renin-angiotensin systems. Am. J. Physiol. Metab..

[18] Kong J., Kim G.H., Wei M., Sun T., Li G., Liu S.Q., Li X., Bhan I., Zhao Q., Thadhani R. (2010). Therapeutic Effects of Vitamin D Analogs on Cardiac Hypertrophy in Spontaneously Hypertensive Rats. Am. J. Pathol..

[19] Rangan G.K., Schwensen K.G., Foster S., Korgaonkar M., Peduto A., Harris D.C. (2013). Chronic effects of dietary vitamin D deficiency without increased calcium supplementation on the progression of experimental polycystic kidney disease. Am. J. Physiol. Physiol..

[20] Geng X., Shi E., Wang S., Song Y. (2020). A comparative analysis of the efficacy and safety of paricalcitol versus other vitamin D receptor activators in patients undergoing hemodialysis: A systematic review and meta-analysis of 15 randomized controlled trials. PLoS ONE.

[21] Torres V.E., Abebe K., Chapman A.B., Schrier R.W., Braun W.E., Steinman T.I., Winklhofer F.T., Brosnahan G., Czarnecki P.G., Hogan M.C. (2014). Angiotensin Blockade in Late Autosomal Dominant Polycystic Kidney Disease. N. Engl. J. Med..

[22] Phillips J.K., Hopwood D., Loxley R.A., Ghatora K., Coombes J.D., Tan Y.S., Harrison J.L., McKitrick D.J., Holobotvskyy V., Arnolda L.F. (2007). Temporal Relationship between Renal Cyst Development, Hypertension and Cardiac Hypertrophy in a New Rat Model of Autosomal Recessive Polycystic Kidney Disease. Kidney Blood Press. Res..

[23] Ta M.H.T., Schwensen K.G., Foster S., Korgaonkar M., Ozimek-Kulik J.E., Phillips J.K., Peduto A., Rangan G.K. (2016). Effects of TORC1 Inhibition during the Early and Established Phases of Polycystic Kidney Disease. PLoS ONE.

[24] Sagar P.S., Zhang J., Luciuk M., Mannix C., Wong A.T.Y., Rangan G.K. (2019). Increased water intake reduces long-term renal and cardiovascular disease progression in experimental polycystic kidney disease. PLoS ONE.

[25] Canberra C.N. (2013). Australian Code for the Care and Use of Animals for Scientific Purposes.

[26] Piao S., Song J., Lim S., Chung B., Choi B., Yang C. (2012). Protective Effect of Paricalcitol on Cyclosporine-Induced Renal Injury in Rats. Transplant. Proc..

[27] Sanchez-Niño M.-D., Bozic M., Córdoba-Lanús E., Valcheva P., Gracia O., Ibarz M., Fernández E., Navarro-González J.F., Ortiz A., Valdivielso J.M. (2012). Beyond proteinuria: VDR activation reduces renal inflammation in experimental diabetic nephropathy. Am. J. Physiol. Physiol..

[28] Keith D.S., Torres V.E., Johnson C.M., Holley K.E. (1994). Effect of Sodium Chloride, Enalapril, and Losartan on the Development of Polycystic Kidney Disease in Han:SPRD Rats. Am. J. Kidney Dis..

[29] Kennefick T.M., Al-Nimri M.A., Oyama T.T., Thompson M.M., Kelly F.J., Chapman J.G., Anderson S. (1999). Hypertension and renal injury in experimental polycystic kidney disease. Kidney Int..

[30] Finch J.L., Tokumoto M., Nakamura H., Yao W., Shahnazari M., Lane N., Slatopolsky E. (2010). Effect of paricalcitol and cinacalcet on serum phosphate, FGF-23, and bone in rats with chronic kidney disease. Am. J. Physiol. Physiol..

[31] Zittermann A., Trummer C., Theiler-Schwetz V., Lerchbaum E., März W., Pilz S. (2021). Vitamin D and Cardiovascular Disease: An Updated Narrative Review. Int. J. Mol. Sci..

[32] Charoenngam N., Holick M.F. (2020). Immunologic Effects of Vitamin D on Human Health and Disease. Nutrients.

[33] Egido J., Martínez-Castelao A., Bover J., Praga M., Torregrosa J.V., Fernández-Giráldez E., Solozábal C. (2016). Efectos pleiotrópicos del paricalcitol, más allá del metabolismo óseo-mineral. Nefrología.

[34] Galuška D., Pácal L., Kaňková K. (2021). Pathophysiological Implication of Vitamin D in Diabetic Kidney Disease. Kidney Blood Press. Res..

[35] Toussaint N.D., Damasiewicz M.J. (2017). Do the benefits of using calcitriol and other vitamin D receptor activators in patients with chronic kidney disease outweigh the harms?. Nephrology.

[36] Vendramini L.C., Dalboni M.A., de Carvalho J.T.G., Batista M.C., Nishiura J.L., Heilberg I.P. (2019). Association of Vitamin D Levels With Kidney Volume in Autosomal Dominant Polycystic Kidney Disease (ADPKD). Front. Med..

[37] Lin L., Zhang L., Li C., Gai Z., Li Y. (2019). Vitamin D and Vitamin D Receptor: New Insights in the Treatment of Hypertension. Curr. Protein Pept. Sci..

[38] Alam A., Perrone R.D. (2013). Left ventricular hypertrophy in ADPKD: Changing demographics. Curr. Hypertens. Rev..

[39] Teng M., Wolf M., Lowrie E., Ofsthun N., Lazarus J.M., Thadhani R. (2003). Survival of Patients Undergoing Hemodialysis with Paricalcitol or Calcitriol Therapy. N. Engl. J. Med..

[40] Tsuruoka S., Wakaumi M., Yamamoto H., Fujimura A. (2004). Chronopharmacology of oxacalcitriol in rat model of osteoporosis. Eur. J. Pharmacol..

[41] Chavhan S., Brar R., Banga H., Sandhu H., Sodhi S., Gadhave P., Kothule V., Kammon A. (2011). Clinicopathological studies on vitamin D3toxicity and therapeutic evaluation of Aloe vera in rats. Toxicol. Int..

[42] Grau L., Gitomer B., McNair B., Wolf M., Harris P., Brosnahan G., Torres V., Steinman T., Yu A., Chapman A. (2020). Interactions between FGF23 and Genotype in Autosomal Dominant Polycystic Kidney Disease. Kidney360.

[43] Lang F., Föller M. (2014). Enigmatic Cassandra: Renal FGF23 formation in polycystic kidney disease. Kidney Int..

[44] Agarwal R. (2010). Are vitamin D receptor agonists like angiotensin-converting enzyme inhibitors without side effects?. Kidney Int..

[45] De Zeeuw D., Agarwal R., Amdahl M., Audhya P., Coyne D., Garimella T., Parving H.-H., Pritchett Y., Remuzzi G., Ritz E. (2010). Selective vitamin D receptor activation with paricalcitol for reduction of albuminuria in patients with type 2 diabetes (VITAL study): A randomised controlled trial. Lancet.

[46] Kuhlmann A., Haas C.S., Gross M.-L., Reulbach U., Holzinger M., Schwarz U., Ritz E., Amann K. (2004). 1,25-Dihydroxyvitamin D3decreases podocyte loss and podocyte hypertrophy in the subtotally nephrectomized rat. Am. J. Physiol. Physiol..

[47] Panizo S., Barrio-Vázquez S., Naves-Díaz M., Carrillo-Lopez N., Rodríguez I., Fernández-Vázquez A., Valdivielso J.M., Thadhani R., Cannata-Andía J.B. (2013). Vitamin D receptor activation, left ventricular hypertrophy and myocardial fibrosis. Nephrol. Dial. Transplant..

[48] Panizo S., Carrillo-Lopez N., Naves-Díaz M., Berrocal G.S., Arias L.M., Díez R.R., Fernández-Vázquez A., Martínez-Salgado C., Ruiz-Ortega M., Dusso A. (2017). Regulation of miR-29b and miR-30c by vitamin D receptor activators contributes to attenuate uraemia-induced cardiac fibrosis. Nephrol. Dial. Transplant..

[49] Leyssens C., Verlinden L., Verstuyf A. (2014). The future of vitamin D analogs. Front. Physiol..

[50] Nakatani S., Nishide K., Okuno S., Ishimura E., Kabata D., Morioka F., Machiba Y., Uedono H., Tsuda A., Shoji S. (2021). Cinacalcet may suppress kidney enlargement in hemodialysis patients with autosomal dominant polycystic kidney disease. Sci. Rep..

