# Supplementary material for: Effect of Early and Delayed Commencement of Paricalcitol in Combination with Enalapril on the Progression of Experimental Polycystic Kidney Disease
Source: J Cardiovasc Dev Dis. 2021 Oct 29;8(11):144. doi: 10.3390/jcdd8110144 (PMC8621425; doi:10.3390/jcdd8110144)
Supplement: Supplementary file 1 [file jcdd-08-00144-s001.zip › jcdd-1388588-supplementary.pdf]

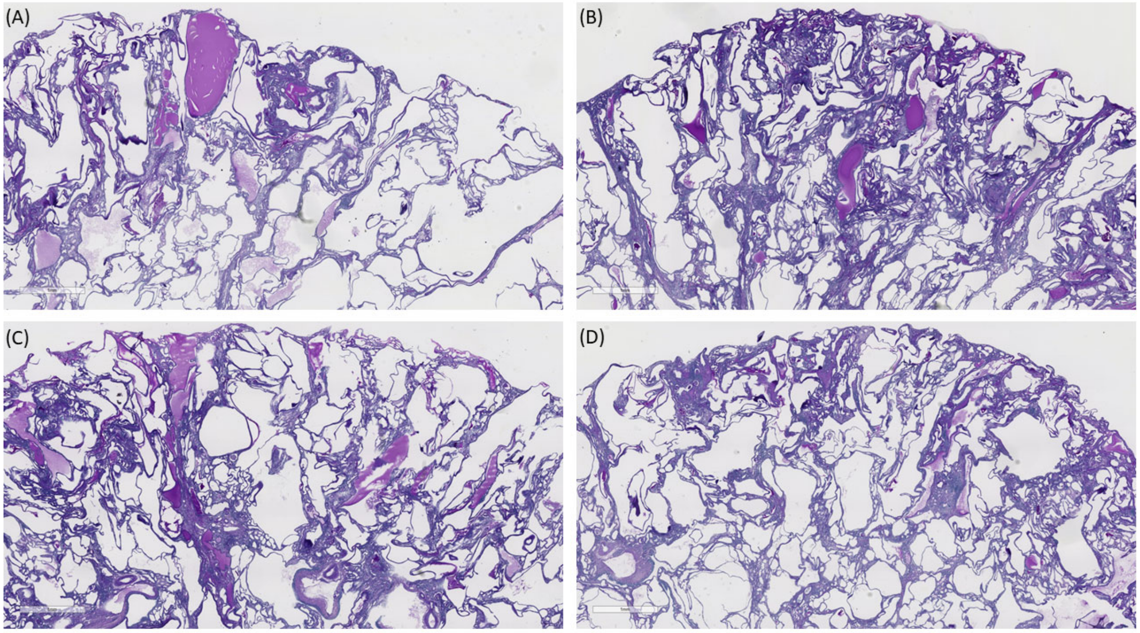

Figure S1. Effect of delayed treatment (weeks 10 to 20) with paricalcitol, enalapril and combination of paricalcitol with enalapril on progression of cystic kidney disease in LPK rats. Shown are representative images of Periodic acid-Schiff staining of kidneys of LPK rats at week 20 following delayed treatment with either: (A) vehicle, (B) paricalcitol, (C) enalapril or (D) combination of paricalcitol and enalapril. There were no differences in renal histology between the treatment groups. Scale bars = 5 mm.

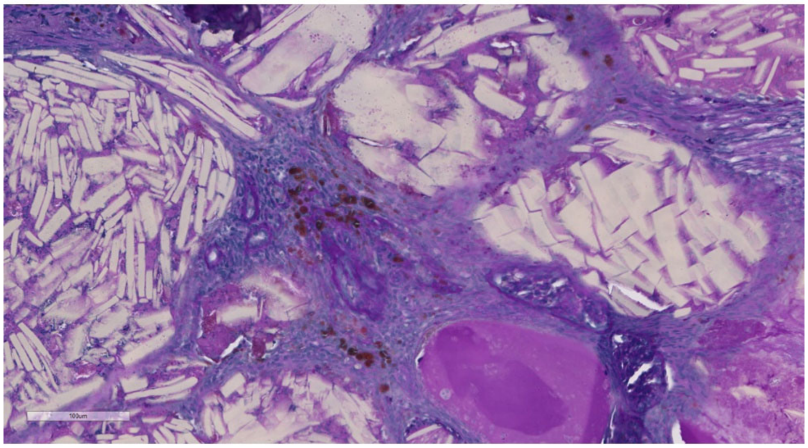

Figure S2. Intra-tubular crystal formation in LPK rats at week 20. Focal areas of intra-tubular crystals were present in all LPK rat groups. A representative example is shown in a LPK rat treated with paricalcitol. Scale bar = 100 μm.

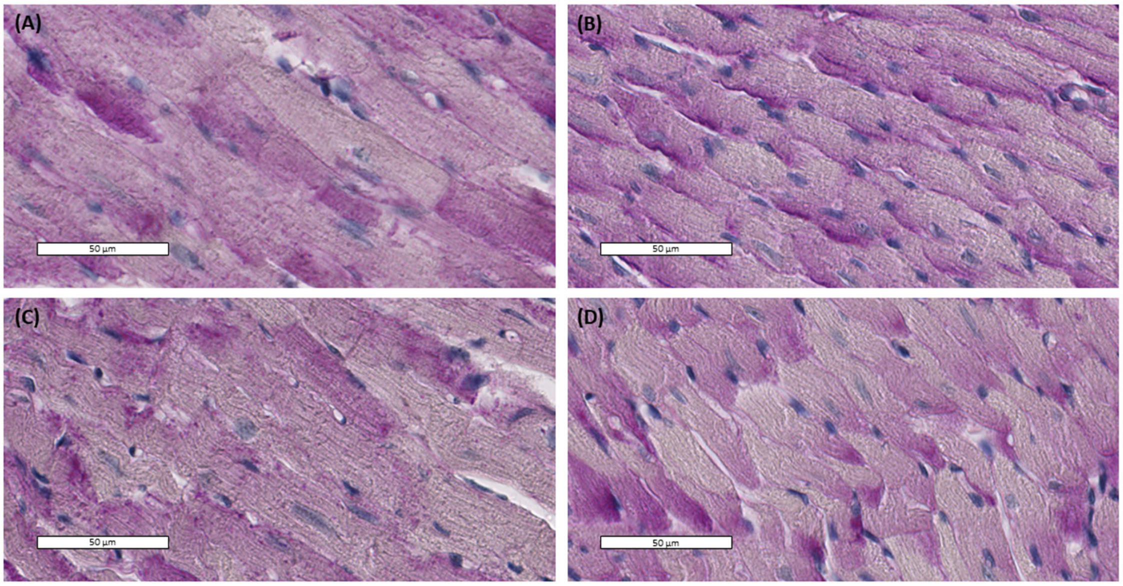

Figure S3. Effect of delayed treatment (weeks 10 to 20) with paricalcitol, enalapril and combination of paricalcitol with enalapril on cardiac histology in LPK rats. Shown are representative images of Periodic acid-Schiff staining of cardiac muscle of LPK rat at week 20 following delayed treatment with (A) vehicle, (B) paricalcitol, (C) enalapril or (D) paricalcitol and enalapril combination starting at week 10. Scale bars = 50 μm.

Table S1: Effect of delayed paricalcitol treatment on body weight (g) over time

Week	Lewis+V (n=6)	LPK+V (n=7)	LPK+PC (n=5)	LPK+E (n=7)	LPK+PC+E (n=7)
10	244.5±16.5	238.42±13.3	241.2±9.7	242.1±19.1	246.6±13.9
11	259±12.6	252.9±17.3	256.3±10.3	256.1±21.4	263.6±17.6
12	282.5±15.8	266.1±19.2	267.7±11.4	265±21.1	264.6±16.8
13	295.8±19.2	276.7±21.5	279.8±10.7	275.9±24.4	282.3±11.6
14	305±15.1	284±21.6	284.7±9.3	283±28.3	291.1±14.7
15	315.2±19.2	295.1±19.8	291.7±6.4	294.9±22.2	296.7±14.1
16	326.8±18.8	300.3±19.4*	292.7±10.1*	302.1±20.1	297.6±16.9
17	333.3±13.9	300.3±20.7*	279.8±17.5*	302±24*	294.1±25.3*
18	339±10.6	310.2±14.8	278.7±12.3*#	300.7±17.4	276.8±17.4*
19	347.3±11.4	310±18.7*	272.7±17.4*#†	308.2±16.9*	269.8±19.3*#†
20	350.7±9.3	312±12.5*	272.4±9.8*#†	323.3±3.8*	256.8±16.1*#†

[1]Abbreviations: LPK, Lewis polycystic kidney rat; V, vehicle; PC, paricalcitol; E, enalapril. Data expressed as mean±standard deviation. *p=<0.05 compared to Lewis+V group, #p<0.05 compared to the LPK+V group. †p<0.05 compared to the LPK+E group.

Table S2: Mean water intake (mls/day) in LPK and Lewis rats over the treatment period in the delayed paricalcitol treatment

Lewis+V (n=6)	LPK+V (n=7)	LPK+PC (n=5)	LPK+E (n=7)	LPK+PC+E (n=7)
22.1±2.8	43.1±5.0*	45.6±4.8*	41.9±4.5*	46.1±6.4*

[1]Abbreviations: LPK, Lewis polycystic kidney rat; V, vehicle; PC, paricalcitol; E, enalapril. Data expressed as mean±standard deviation. *p<0.01 compared to Lewis+V;

Table S3: Effect of delayed paricalcitol treatment on urine volume (mls) in LPK rats.

Week	Lewis+V (n=6)	LPK+V (n=7)	LPK+PC (n=5)	LPK+E (n=7)	LPK+PC+E (n=7)
13	5±2	14±7*	14±5*	15±2*	*13±3**
16	5±2	22±3*	*21±7**	*19±3**	*22±6**
19	4±1	*17±4**	*18±4**	*19±3**	*19±5**[1]

[1]Abbreviations: LPK, Lewis polycystic kidney rat; V, vehicle; PC, paricalcitol; E, enalapril. Data expressed as mean±standard deviation. *p<0.01 compared to Lewis+V

Table S4: Effect of delayed paricalcitol treatment on urinary calcium excretion (urine calcium:creatinine ratio) in LPK rats.

Week	Lewis+V (n=6)	LPK+V (n=7)	LPK+PC (n=5)	LPK+E (n=7)	LPK+PC+E (n=7)
13	0.09±0.03	0.27±0.06*	0.30±0.08*	0.25±0.05*	0.25±0.08*
16	0.10±0.08	0.22±0.05	0.33±0.10*	0.21±0.03	0.27±0.11*
19	0.13±0.07	0.32±0.06*#	0.44±0.05*	0.29±0.08*#	0.34±0.09*[1]

[1]Abbreviations: LPK, Lewis polycystic kidney rat; V, vehicle; PC, paricalcitol; E, enalapril. Data expressed as mean±standard deviation. *p<0.01 compared to Lewis+V; #p<0.05 compared to LPK+PC.

Table S5: Effect of delayed paricalcitol treatment on the progression of proteinuria in LPK rats

Week	Lewis+V (n=6)	LPK+V (n=7)	LPK+PC (n=5)	LPK+E (n=7)	LPK+PC+E (n=7)
13	12.1±19.1	277.7±42.8*	281.2±64.7*	147.4±17.5*#	277.5±145.8*
16	12.5±13.1	477.2±179.0*	448.9±82.6*	221.2±58.8*#	265.1±102.7*#
19	3.5±4.4	1129.4±316.3*	663.7±287.9*#	350.0±165.2*#	296.4±137.4*#[1]

[1]Abbreviations: LPK, Lewis polycystic kidney rat; V, vehicle; PC, paricalcitol; E, enalapril. Data expressed as mean±standard deviation. *p<0.01 compared to Lewis+V; #p<0.05 compared to LPK+V.

Table S6: Effect of delayed paricalcitol treatment on the progression of systolic blood pressure in LPK rats

Week	Lewis+V (n=6)	LPK+V (n=7)	LPK+PC (n=5)	LPK+E (n=7)	LPK+PC+E (n=7)
13	81±5	128±15*	138±11*	104±17*#†	100±6*#†
16	73±5	124±26*	123±17*	109±12*	103±12
19	77±4	119±18*	127±10*	117±12*	94±16#†‡

[1]Abbreviations: LPK, Lewis polycystic kidney rat; V, vehicle; PC, paricalcitol; E, enalapril. Data expressed as mean±standard deviation. *p<0.05 compared to Lewis+V; #p<0.05 compared to LPK+V. †p<0.01 compared to LPK+PC. ‡p<0.01 compared to LPK+E.
